# Partial androgen insensitivity syndrome caused by a deep intronic mutation creating an alternative splice acceptor site of the *AR* gene

**DOI:** 10.1038/s41598-018-20691-9

**Published:** 2018-02-02

**Authors:** Hiroyuki Ono, Hirotomo Saitsu, Reiko Horikawa, Shinichi Nakashima, Yumiko Ohkubo, Kumiko Yanagi, Kazuhiko Nakabayashi, Maki Fukami, Yasuko Fujisawa, Tsutomu Ogata

**Affiliations:** 1 Department of Pediatrics, Hamamatsu University School of Medicine, Hamamatsu, Japan; 2 Department of Biochemistry, Hamamatsu University School of Medicine, Hamamatsu, Japan; 3 Division of Endocrinology and Metabolism, National Medical Center for Children and Mother, Tokyo, Japan; 4 Department of Pediatrics, Shizuoka Saiseikai Hospital, Shizuoka, Japan; 5 Department of Genome Medicine, National Research Institute for Child Health and Development, Tokyo, Japan; 6 Department of Maternal-Fetal Biology, National Research Institute for Child Health and Development, Tokyo, Japan; 7 Department of Molecular Endocrinology, National Research Institute for Child Health and Development, Tokyo, Japan

## Abstract

Although partial androgen insensitivity syndrome (PAIS) is caused by attenuated responsiveness to androgens, androgen receptor gene (*AR*) mutations on the coding regions and their splice sites have been identified only in <25% of patients with a diagnosis of PAIS. We performed extensive molecular studies including whole exome sequencing in a Japanese family with PAIS, identifying a deep intronic variant beyond the branch site at intron 6 of *AR* (NM_000044.4:c.2450−42 G > A). This variant created the splice acceptor motif that was accompanied by pyrimidine-rich sequence and two candidate branch sites. Consistent with this, reverse transcriptase (RT)-PCR experiments for cycloheximide-treated lymphoblastoid cell lines revealed a relatively large amount of aberrant mRNA produced by the newly created splice acceptor site and a relatively small amount of wildtype mRNA produced by the normal splice acceptor site. Furthermore, most of the aberrant mRNA was shown to undergo nonsense mediated decay (NMD) and, if a small amount of aberrant mRNA may have escaped NMD, such mRNA was predicted to generate a truncated AR protein missing some functional domains. These findings imply that the deep intronic mutation creating an alternative splice acceptor site resulted in the production of a relatively small amount of wildtype *AR* mRNA, leading to PAIS.

## Introduction

Partial androgen insensitivity syndrome (PAIS) is a rare endocrine disorder caused by attenuated responsiveness to androgens^1^. Affected 46,XY patients frequently exhibit undermasculinized genitalia since birth and gynecomastia with puberty, in the presence of age-appropriate serum androgen values^1^. Testis development is usually normal, while small testes have been identified in a substantial fraction of pubertal or adult patients with molecularly confirmed PAIS^2^.

Notably, androgen receptor gene (*AR*) mutations on the coding regions and their splice sites have been identified only in <25% of patients with a clinical diagnosis of PAIS, even after excluding other representative causes for PAIS-like phenotypes^1,3^. Furthermore, such *AR* mutations remain undetected in a certain fraction of patients with compromised endogenous AR activity^4^. These findings imply the possible presence of hidden mutations in non-coding regions of *AR* or in other genes involved in AR-signaling. Here, we report a deep intronic mutation creating an alternative splice acceptor site at intron 6 of *AR*.

## Results

### Clinical report

**Figure 1:**
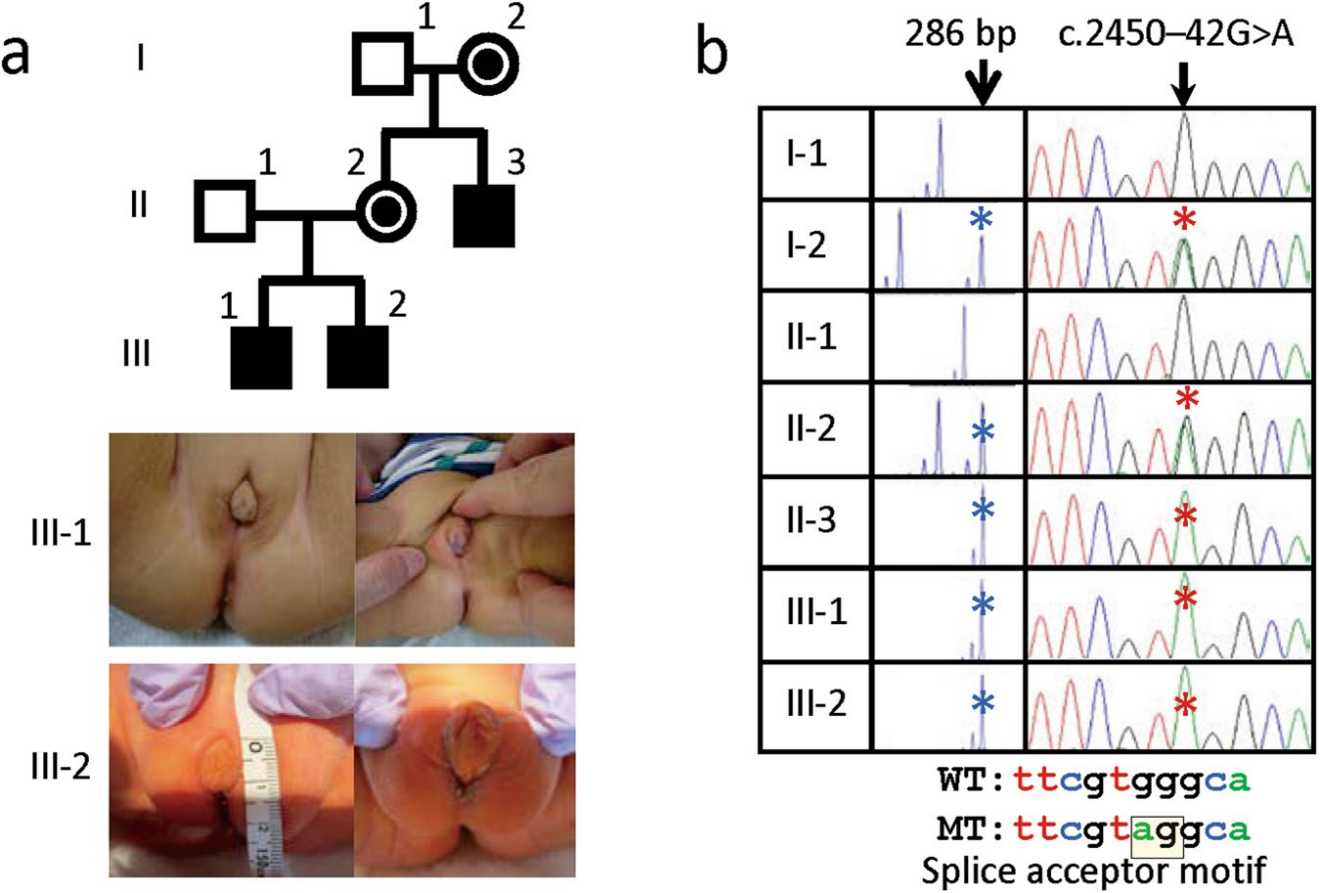
Clinical and genetic findings of this family. (**a**) The pedigree of this family, and external genital findings of the affected boys (III-1 and III-2). (**b**) CAG repeat length polymorphism at exon 1 of *AR* and c.245–42 G > A variant at intron 6 of *AR*. The “286 bp” peak and the “A” allele are shared by affected males II-3, III-1, and III-2, and obligatory carriers I-2 and II-2. The c.245–42 G > A variant creates the “AG” splice acceptor motif. WT: wildtype, and MT: mutant.

We encountered a Japanese family with an X-linked recessive or sex-limited autosomal dominant form of 46,XY disorder of sex development (DSD) (Fig. 1a). The proband (II-3) was referred to us because of undermasculinized genitalia at 18 years of age. Subsequently, two similarly affected boys (III-1 and III-2) were born to his elder sister (II-2). The two obligatory carrier females (I-2 and II-2) were clinically normal, as were their non-consanguineous husbands (I-1 and II-1).

Clinical findings of the three affected males are summarized in Table 1. They exhibited undermasculinized genitalia including penoscrotal hypospadias and microphallus since birth (Fig. 1a). Patient II-3 also manifested gynecomastia with puberty, and small testes at 18 years of age. Müllerian derivatives were absent. Endocrine studies revealed sufficient androgen production capacities and hypergonadotropinism. Surgical treatment for undermasculinized genitalia and/or gynecomastia, and penile elongation therapy with testosterone enanthate and dihydrotestosterone cream, were performed or planned for the three affected males. Penile length responses to elongation therapy remained quite poor in patients II-3 and III-1. Thus, the three affected males were diagnosed as having PAIS.

**Table 1:** Clinical findings of three patients examined in this study

Patient	II-3	III-1	III-2
Karyotype	46,XY	46,XY	46,XY
Social sex	Male	Female → Male^a^	Male
Present age	27 y 2 m	2 y 6 m	0 y 4 m
<**Genital findings**>			
Age at examination	Infancy	6 m	1 m
Tanner stage	PH 1, G 1	PH 1, G 1	PH 1, G 1
Testis size (mL)	2 (Bilateral)	2 (Bilateral)	2 (Bilateral)
Hypospadias	+(Penoscrotal)	+(Penoscrotal)	+(Penoscrotal)
Cryptorchidism	+(Bilateral)	+(Bilateral)	—
Microphallus (PL, cm)	+(*~2.0*)	+(~*2.3*)	+(~1.2)
Abnormal scrotum	+(Bifid)	+(Hypoplastic)	+(Bifid)
Uterus/Vagina	…	Absent on MRI	Absent on MRI
Age at examination	18 y 11 m		
Tanner stage	PH 5, *G 3* (after Tx)^b^		
Testis size (mL)	*5* (Right), *3–4* (Left)		
Microphallus (PL, cm)	+(*~2.8*) (after Tx)^b^		
Uterus/Vagina	Absent on MRI		
<**Endocrine findings**>			
Age at examination	14 y 6 m	6 m	1 m
LH (IU/L)	**12.8** → **58.1**^c^	1.9	0.4
FSH (IU/L)	**24.0** → **35.6** ^c^	2.8	1.1
Testosterone (nmol/L)	16.1 → 18.3^d^	5.9	2.4 → 13.7^d^
DHT (nmol/L)	0.9 → 1.0^d^	1.9	0.6 → 2.3^d^
Age at examination	18 y 11 m	10 m	3 m
LH (IU/L)	**37.5**	2.1 → **47.4**^c^	1.6
FSH (IU/L)	**17.7**	2.6 → 8.4^c^	1.2
Testosterone (nmol/L)	18.4	8.8 → 30.4^d^	9.8
DHT (nmol/L)	1.3	3.6 → 3.7^d^	…
<**Treatment**>			
Orchidopexy (age)	Performed (5 y)	Performed (16 m)	Planned
Genitoplasty (age)	Performed (5 y)	Planned	Planned
Mastectomy (age)	Performed (14 y, 16 y)	…	…
TE (25 mg i.m.) (age)	>5× (14 y)	6× (13–24 m)	Planned
DHT cream (age)	>Six months (15 y)	Two months (2 y)	
PL increment (cm)	~2.0 → ~2.8^e^	~2.3 → ~2.8^e^	
<**Gender role**>	Male^f^		

Abbreviations: PL, penile length; LH, luteinizing hormone; FSH, follicle stimulating hormone; DHT, dihydrotestosterone; TE, testosterone enanthate; y, year; m, month; PH, pubic hair; G, genitalia; Tx, treatment; and MRI, magnetic resonance imaging.

The data below the age-matched reference values^19–22^ are italicized and underlined, whereas those above the age-matched reference values^19–22^ are boldfaced and underlined.

^a^Social sex was changed from female to male at 12 months of age after detailed examinations and thorough consultation.

^b^Treatment with TE injection and topical DHT cream, as described below.

^c^Basal and peak values during a gonadotropin releasing hormone stimulation test (100 µg/m^2^ [max. 100 µg] bolus i.v.; blood sampling at 0, 30, 60, 90, and 120 min).

^d^Basal and stimulated values in a human chorionic gonadotropin stimulation test (3000 IU/m^2^/dose [max. 5000 IU] i.m. for three consecutive days; blood sampling on days 1 and 4).

^e^Penile length response to TE (25 mg i.m.) in prepubertal boys with hypospadias who are free from demonstrable *AR* and *SRD5A2* mutation is 0.35 cm per dose^23^.

^f^This patient is living with a female partner.

### Comprehensive sequencing analyses of exons and their splice sites

We first performed Sanger direct sequencing for *AR* and for *NR5A1*, *MAMLD1*, and *SRD5A2* whose mutations often cause PAIS-like phenotypes^5^ in the three affected males, identifying no pathogenic sequence variant on the coding exons and their splice sites. We next analyzed 25 hypospadias-related genes by target enrichment system^6,7^, revealing no pathogenic variant on the coding exons and their splice sites shared by the three affected males.

Thus, we carried out whole exome sequencing in all the seven subjects of this family. However, no pathogenic variant co-segregating with the PAIS phenotype was identified on the coding exons and their splice sites (~10 bp of intronic sequences) of all the previously known genes involved in hypospadias or DSD^1,6–8^. Furthermore, although a total of 20 autosomal, but not X-linked, gene variants (variants 1–20 in Supplementary Table S1) with minor allele frequencies (MAFs) of ≤0.01 in all the four public databases and in-house database were found to be present in the three affected males (II-3, III-1, and III-3) and the two carrier females (I-2 and II-2) and absent from the two non-affected males (I-1 and II-1), they were assessed as apparently irrelevant to the PAIS phenotype on the basis of the frequencies in the normal populations, the results of *in silico* pathogenic analyses, and/or the available information regarding the corresponding genes. Indeed, while missense variants 2, 4, and 15 were completely absent from the public and in-house databases, variants 4 and 15 were evaluated as non-pathogenic by ≥ 3 of the four *in silico* analyses. Similarly, while missense variants 1, 2, and 12 were evaluated as pathogenic by all the four *in silico* analyses, variants 1 and 12 were, though extremely rare, registered in the public databases, as were a nonsense variant 5 and a splice donor site variant 14. Furthermore, while variant 2 was completely absent from the public and in-house databases and was assessed as pathogenic by all the four *in silico* analyses, the tissue expression pattern of the corresponding gene (*SOAT1*) was apparently inconsistent with the PAIS phenotype.

### Genomewide array comparative genomic hybridization (aCGH)

We also performed aCGH in the seven subjects in this family, to examine the presence or absence of a copy-number variant as an underlying factor for PAIS. However, aCGH identified no pathologic copy number variant co-segregating with the PAIS phenotype in this family.

### **Identification of a deep intronic variant in*AR***

Since no definitive variant was detected by the above studies, we suspected that a pathogenic variant might be hidden in non-coding regions of *AR*. In support of this, the analysis of CAG repeat length polymorphism on exon 1 of *AR* showed co-segregation of the same allele with the PAIS phenotype (Fig. 1b). Thus, we carefully searched *AR* non-coding regions covered by the whole exome sequencing for a rare variant (MAFs of ≤ 0.01 in the four public databases and in-house database shown in Supplementary Table S1) co-segregating with the PAIS phenotype. Consequently, only a single variant, i.e., a deep intronic variant beyond the branch site at intron 6 (NM_000044.4:c.2450−42 G > A) creating a new “AG” splice acceptor motif (https://www.ncbi.nlm.nih.gov/genbank/) was found to be present in the three affected males and the two carrier females and absent from the two non-affected males, as shown by Sanger sequencing (Fig. 1b). This variant was completely absent from the four public databases and in-house database.

### ***In silico*analyses for the deep intronic variant**

**Figure 2:**
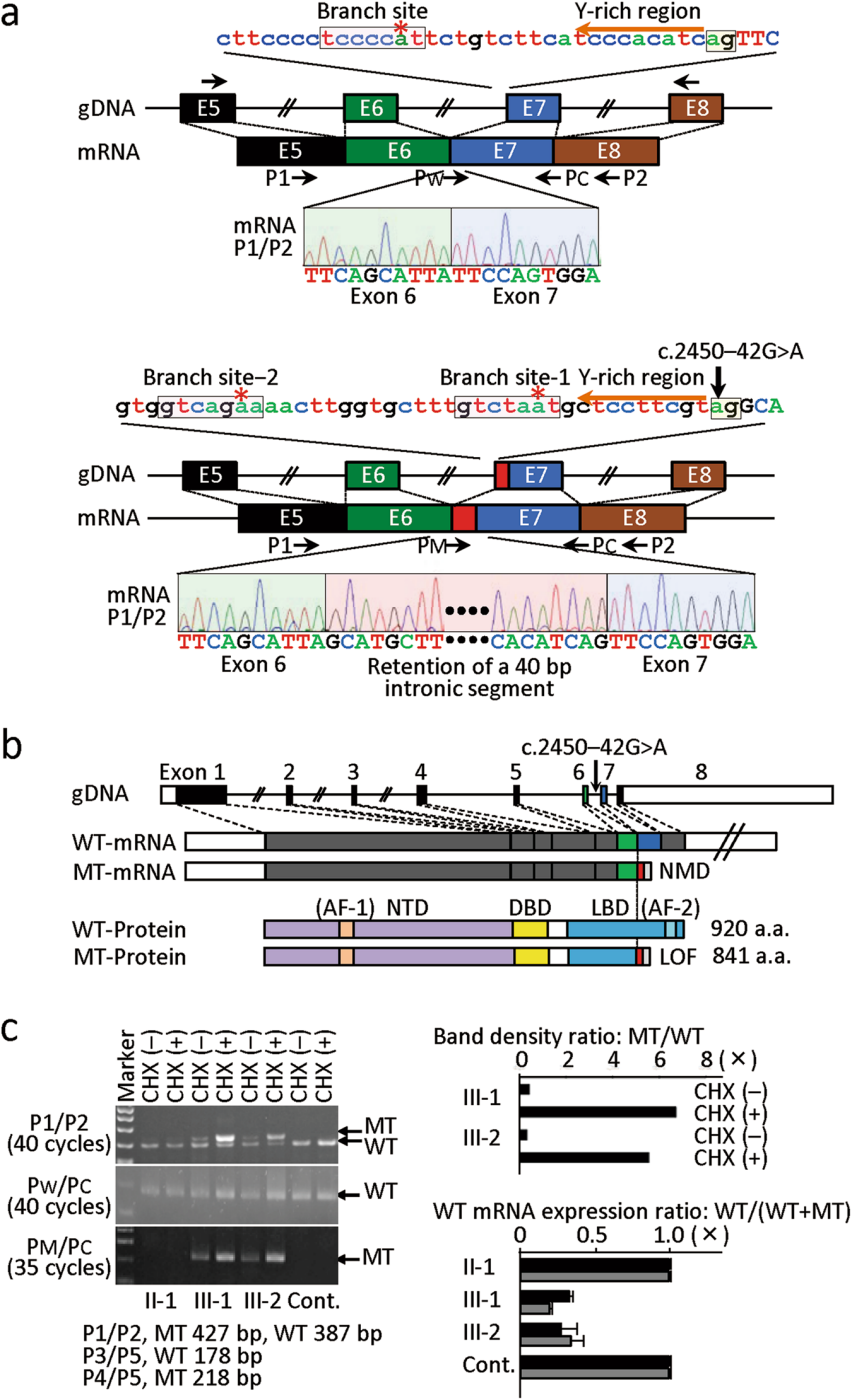
Alternative splicing caused by the c.245–42 G > A variant creating the “AG” splice acceptor site. (**a**) Normal splicing identified in the affected boys (III-1 and III-2). Normal splicing has occurred using the wildtype “AG” splice acceptor motif, pyrimidine (Y)-rich sequence, and the branch site, generating wildtype mRNA, whereas aberrant splicing has taken place using the newly created “AG” splice acceptor motif that is associated with pyrimidine (Y)-rich sequence and two putative branch sites, producing mRNA with retention of a 40 bp intronic segment (highlighted with red) between exon 6 (highlighted with green) and exon 7 (highlighted with blue). Red asterisks indicate the highly conserved “A” nucleotide at branch sites. Exonic and transcribed intronic nucleotides are written with large letters, and intronic nucleotides with small letters. (**b**) Schematic representation of *AR* exons on the genome, and wildtype (WT) and mutant (MT) mRNA and protein. AF-1: activation function 1; NTD: N-terminal transactivation domain; DBD: DNA binding domain; LBD: ligand binding domain; and AF-2: activation function 2. The coding regions are shown with solid boxes, and the untranslated regions are depicted with open boxes; exon 6, exon 7, the transcribed intronic sequence, and the frameshifted sequence before the premature termination are shown in green, blue, red, and gray, respectively. It is predicted that the mutant mRNA primarily undergoes nonsense mediated decay (NMD), while it could escape NMD, producing a small amount of a truncated protein with a loss-of-function (LOF) activity. (**c**) RT-PCR analysis for mRNA samples extracted from the LCLs of the non-affected father (II-1), the affected boys (III-1 and III-2), and a healthy control adult male. The band intensity ratio has been obtained between the wildtype (WT)-specific and the mutant (MT)-specific bands produced by the P1/P2 primers in the affected boys. The WT mRNA expression ratio has been calculated with the data of quantitative PCR performed for CHX-treated LCLs using P_W_/Pc or P_M_/Pc; the black and the gray bars have been obtained using *GAPDH* and *ACTB* as internal controls, respectively.

Thus, we examined whether the new “AG” splice acceptor motif is accompanied by a pyrimidine (Y)-rich sequence and a branch site required for splicing^9^, as for the wildtype “AG” splice acceptor site at intron 6 (Fig. 2a). Human Splicing Finder (www.umd.be/HSF3/HSF.html) indicated the presence a pyrimidine-rich sequence and two candidate branch sites near the newly created “AG” splice acceptor motif (Fig. 2a). Furthermore, it was predicted that aberrant mRNA produced by the new “AG” motif undergoes nonsense-mediated decay (NMD)^10^ due to retention of a 40-bp intronic sequence and resultant occurrence of premature termination at the 842th codon on exon 7. In addition, if a small amount of aberrant mRNA could escape NMD, such mRNA was predicted to generate a truncated AR protein missing some functional domains (Fig. 2b).

### **Expression studies of the wildtype and aberrant*AR*mRNAs**

We further studied whether an alternative splicing actually occurs using the newly created “AG” splice acceptor site. For this purpose, we performed reverse-transcriptase (RT)-PCR analysis, using Epstein-Barr virus (EBV)-transformed B-cell lymphoblastoid cell lines (LCLs) established from the affected boys (III-1 and III-2), their 30-year-old non-affected father (II-1), and a 28-year-old healthy adult male with proven fertility. The mRNA samples were obtained after 8-hour incubation with or without the NMD inhibitor cycloheximide (CHX).

RT-PCR analysis was performed with primers which could amplify both the wildtype mRNA and the predicted aberrant mRNA (P1 primer for exon 5; and P2 primer for exon 8) (Fig. 2a). Consequently, 387 bp and 427 bp products were identified in the affected boys (III-1 and III-2), whereas a 387 bp product alone was detected in the non-affected father (II-1) and the control male (Fig. 2c). There was no other RT-PCR product, such as that formed with skipping of exon 6 or exon 7. While the band intensity of the 387 bp product was grossly similar between CHX-untreated and CHX-treated LCLs of each subject, that of the 427 bp product was obviously stronger for CHX-treated LCLs than for CHX-untreated LCLs of the affected boys (III-1 and III-2), with the band intensity ratio between the 387 bp and 427 bp products obtained from CHX-treated LCLs being ~1:6. Sequencing of the two RT-PCR products showed that the 387 bp product was derived from the normally spliced wildtype mRNA, whereas the 427 bp product was derived from the aberrant mRNA with retention of a 40-bp intronic sequence, as predicted (Fig. 2a).

RT-PCR analysis was also carried out with primers (P_W_/Pc) designed to amplify the wildtype mRNA alone and those (P_M_/Pc) designed to amplify the mutant mRNA alone (P_W_: a primer specific to the wildtype splice junction between exon 6 and exon 7; P_M_: a primer specific to the mutant splice junction between exon 6 and retained intron 6; and P_C_: a primer for the splice junction between exon 7 and exon 8 commonly utilized for the wildtype and mutant mRNA amplifications) (Fig. 2a). The band intensity for the wildtype mRNA was grossly similar between CHX-untreated and CHX-treated LCLs of the four subjects examined, whereas that for the aberrant mRNA was weak for the CHX-untreated LCLs and strong for the CHX-treated LCLs of the affected boys (III-1 and III-2) (Fig. 2c). Furthermore, quantitative RT-PCR was performed with the P_W_/Pc and P_M_/Pc primers, using *GAPDH* or *ACTB* as an internal control, indicating that the wildtype mRNA expression ratio (the ratio between the wildtype mRNA expression dosage and the sum of the wildtype and aberrant mRNA expression dosages obtained from CHX-treated LCLs) was 25–30% in the affected boys and ~100% in the non-affected male and the control male (Fig. 2c). However, precise comparisons of the wildtype or aberrant mRNA expression dosage among different subjects was impossible, because of variable expression dosages among subjects. For example, the sum of the wildtype and aberrant mRNA expression dosage obtained from CHX-treated LCLs was apparently large in the affected boy (III-1) and small in the non-affected male (II-1).

## Discussion

We identified a deep intronic mutation (c.2450−42 G > A) creating an alternative splice acceptor site at intron 6 of *AR* in a family with PAIS. This new acceptor site was accompanied by a pyrimidine-rich sequence and two candidate branch sites required for splicing and, consistent with this, RT-PCR experiments for CHX-treated LCLs revealed a relatively large amount of aberrant mRNA produced by the newly created alternative splice acceptor site and a relatively small amount of wildtype mRNA yielded by the original normal splice acceptor site. Furthermore, most of the aberrant mRNA was shown to undergo NMD and, if a small amount of aberrant mRNA may have escaped NMD *in vivo*, as suggested by the weak but discernible bands for the aberrant mRNA obtained from CHX-untreated LCLs of the affected boys (III-1 and III-2), such mRNA was predicted to generate a truncated AR protein missing some functional domains. These findings imply that the deep intronic mutation has resulted in the production of a relatively small amount of wildtype *AR* mRNA, leading to PAIS.

To our knowledge, three intronic aberrations have been identified in *AR*, except for splice site mutations at the conserved “GT-AG” motif: [1] a > 6 kb deletion at intron 2 involving a putative branch site, which produced ~90% of aberrant mRNA encoding non-functional AR protein due to in-frame skipping of exon 3 and ~10% of wildtype mRNA produced by a cryptic branch site, in a family with typical PAIS^11^; [2] c.1769−11 T > A at the pyrimidine-rich region at intron 2, which produced two types of aberrant mRNA encoding non-functional AR proteins due to in-frame retention of 69-bp intronic sequence transcribed by a cryptic splice acceptor site and in-frame skipping of exon 3, as well as a very low level of wildtype mRNA, in a family with apparently complete AIS (CAIS)^12^ (though described as PAIS in the original paper, the combination of normal female external genitalia and Wolffian development is frequently observed in CAIS with a residual AR activity)^1^; and [3] c.2450−118 A > G at intron 6, which produced two types of mRNA subject to NMD due to retention of 85 bp and 202 bp sequences of intron 6 respectively, in siblings with CAIS^13^. These findings, together with the present data, indicate that several types of intronic mutations other than splice site mutations at the conserved “GT-AG” motif do exist in *AR*, although they remain extremely rare, and that PAIS takes place when a relatively small amount of wildtype *AR* mRNA is produced. In this regard, it would be worth pointing out that the c.2450−42 G > A identified in this study is the first PAIS-causing deep intronic mutation beyond the branch site.

Two points should be made with regard to the present study. First, although we performed extensive molecular studies, possible relevance of other variant(s) has not been excluded formally. Indeed, a true pathogenic variant for 46,XY DSD might have been overlooked by the whole exome sequencing, if it has been registered in the public and/or in-house databases because of its detection in normal females or in the general population due to incomplete penetrance, or if its pathogenic assessment remains more or less equivocal. In addition, such a variant might be hidden in non-examined sequences of *AR* or other genes.

Second, precise comparison of expression dosage between different subjects was impossible even by the quantitative RT-PCR, because of variable *AR* mRNA expressions among subjects. Thus, it remains to be demonstrated whether the wildtype mRNA expression dosage is actually reduced in the affected males as compared with that in the non-affected males, although the present study revealed markedly reduced relative wildtype mRNA expression ratios in the affected males. This would primarily be due to expression studies being performed for polyclonal EBV-transformed LCLs that could express multiple genes to highly variable degrees, probably depending on the character of clones examined^14^. For example, it is possible that the LCLs of the affected boy III-1, which showed a high level of *AR* expression, were primarily composed of clones that efficiently express *AR*. In addition, since we utilized LCLs of non-affected adult father (II-1) and a healthy adult male for controls primarily because of an ethical issue, the age difference between the affected boys and the control adults may also be relevant to the inter-individual difference in the *AR* expression dosage.

Despite such caveats, the present data strongly support that the deep intronic mutation of *AR* (c.2450−42 G > A) is the pathogenic variant for PAIS. Further studies will permit to clarify the relevance of *AR* intronic mutations to the development of PAIS and CAIS.

## Methods

### Ethical approval

This study was approved by the Institutional Review Board Committee at Hamamatsu University School of Medicine, and performed after obtaining written informed consent to participate in this study and to publish molecular and clinical data including external genital findings/images from the adult subjects or from the parents of the affected children. All experiments were performed in accordance with the relevant guidelines and regulations.

### Reference sequenc**e**

Of plural *AR* isoforms, transcript variant 1 (NM_000044.4) consisting of eight exons and encoding 920 amino acids was utilized as a reference (GenBank, https://www.ncbi.nlm.nih.gov/genbank/), as suggested previously^15^.

### Primers

Primers utilized in this study are shown in Supplementary Table S2.

### Molecular studies using leukocyte genomic DNA samples

Genomic DNA was obtained from peripheral blood leukocytes using FlexiGene DNA Kit (QIAGEN).

Sanger direct sequencing for *AR*, *NR5A1*, *MAMLD1*, and *SRD5A2* was performed from both directions on the ABI 3130xl Genetic Analyzer (Thermo Fisher Scientific).

Sequence analysis for 25 causative or candidate genes for hypospadias, i.e., *AR*, *ATF3*, *BMP4*, *BMP7*, *BNC2*, *CTGF*, *CYP1A1*, *CYR61*, *DGKK*, *EGF*, *ESR1*, *ESR2*, *FGF8*, *FGFR2*, *GSTM1*, *GSTT1*, *HOXA4*, *HOXB6*, *HSD3B2*, *HSD17B3*, *MAMLD1*, *MID1*, *NR5A1*, *SRD5A2*, and *WT1*^6,7^, was performed using the Haloplex Target Enrichment System (Design ID 10380-1414463301) (Agilent Technologies). After amplification, the coding regions of the 25 genes were sequenced as 150 bp paired-end reads on a MiSeq sequencer (Illumina). Subsequently, nucleotide alterations in the samples were called by the Genome Analysis Toolkit (Broad Institute) and SAMtools 0.1.17 software (http://samtools.sourceforge.net)^16^.

Whole exome sequencing was carried out, using SureSelect Human All Exon V6 (Agilent Technologies). Captured libraries were sequenced by NextSeq. 500 (Illumina) with 150 bp paired-end reads. Reads were aligned to the reference genome (Human GRCh37/hg19) (UCSC Genome Browser; http://genome.ucsc.edu/) using BWA-MEM (Version 0.7.12) with default parameters. Duplicated reads are removed by Picard (Version 1.106), and local realignment and base quality recalibration were performed by GATK Version 3.5. Variants were identified with the GATK HaplotypeCaller, and variants with MAFs > 1% in at least one of four public databases and in-house database shown in Supplementary Table S1 were excluded. Final variants were annotated with Annovar^17^.

Genomewide array comparative genomic hybridization was performed with a catalog human array (1 × 1 M format, ID G4447A) (Agilent Technologies), using leukocyte genomic DNA from sex-matched normal subjects as controls. For autosomes and female X chromosomes, log2 signal ratios of <−0.8 and >+ 0.4 were regarded as indicative of heterozygous deletions and duplications, respectively. For male sex chromosomes that appear in a heterogametic condition, log2 signal ratios of −∞ and around +1.0 were interpreted as hemizygous deletions and duplications, respectively. When ≥three consecutive probes showed abnormal log2 ratios, the corresponding region was regarded as copy number variants (CNVs). The genomic position was based on human GRCh37/hg19. CNVs were regarded as normal variants if they have been registered in the public databases such as Database of Genomic Variants (http://dgv.tcag.ca/dgv/app/home) and ClinVar (http://www.ncbi.nlm.nih.gov/clinvar/).

CAG repeat length on exon 1 of *AR* was determined for PCR products obtained with a fluorescently labeled forward primer and an unlabeled reverse primer on the ABI 3130xl Genetic Analyzer, using GeneMapper Software 5.

### Molecular studies using lymphoblastoid cell line-derived mRNA

EBV transformed LCLs were established from the non-affected male (II-1), the affected boys (III-1 and III-2), and an adult healthy control male with proven fertility by a standard method^18^ with minor modifications, after obtaining written informed consent from the parents of the affected boys and from the non-affected father and the healthy adult male. Because of the ethical issue, we could not establish an LCL from age-matched healthy boy or utilize LCLs established from boys with congenital disorders other than 46,XY DSD.

Total RNA samples were isolated from immortalized LCLs cultured in media containing dimethyl sulfoxide or cycloheximide (Sigma) for 8 hours, using RNeasy Mini Kit (QIAGEN). RT-PCR with P1/P2 primers was performed with one μg of total RNA, using ReverTra Ace qPCR RT Kit (TOYOBO). Then, RT-PCR products were subjected to electrophoresis, and the band intensity was measured with ImageJ version 1.49 software (http://www.imagej.com-about.com/). The RT-PCR products were also subjected to direct sequencing. RT-PCR with P_W_/Pc or P_M_/Pc primers was similarly carried out with one μg of total RNA. Quantitative RT-PCR with P_W_/Pc or P_M_/Pc primers was also performed for mRNA samples extracted from CHX-treated LCLs by the SYBR Green methods on StepOnePlus system with Software v2.2.2 (Thermo Fisher Scientific), using *GAPDH* and *ACTB* as internal controls. We calculated the relative mRNA expression of each sample based on its threshold cycle (Ct) in comparison to the Ct of *GAPDH* or *ACTB*. Then, we calculated the ratio of the wildtype *AR* mRNA expression to the total (the sum of wildtype and mutant mRNAs) *AR* mRNA expression obtained from CHX-treated LCLs.

## Electronic supplementary material

Supplementary information
