# Supplementary material for: Partial androgen insensitivity syndrome caused by a deep intronic mutation creating an alternative splice acceptor site of the AR gene
Source: Sci Rep. 2018 Feb 2;8:2287. doi: 10.1038/s41598-018-20691-9 (PMC5797100; doi:10.1038/s41598-018-20691-9)
Supplement: Supplementary file 1 — Supplementary information [file 41598_2018_20691_MOESM1_ESM.pdf]

Supplementary Table S1. Variants present in three affected males and two caeeier females and absent from two unaffected males in this family.

Identified variant			Minor allele frequency in public database					*In silico* pathogenic analyses				Gene information		
	Variant	Gene	gnomAD_genome_EAS	gnomAD_exome_EAS	HGVD	2KJPN	In-house	CADD (PHRED score)	PP2_HDIV (score)	SIFT (score)	MutationTaster (score)	Phenotype (OMIM)	Inheritance	High expression (UCSC)
1	NM_152367:c.61C>T<br>p.(R21C)	*MAB21L3*<br>(Chr. 1p13.1)	0	0.0002901	0	0	0	1% most deleterious<br>25.3	Probably damaging<br>0.999	Damaging<br>0.01	Disease causing<br>0.968			Esophageal mucosa, vagina, bladder
2	NM_003101:c.359A>G<br>p.(K120R)	*SOAT1*<br>(Chr. 1q25.2)	0	0	0	0	0	1% most deleterious<br>27.0	Probably damaging<br>0.999	Damaging<br>0.01	Disease causing<br>1			Adrenal gland
3	NM_020950:c.1705A>C<br>p.(M569L)	*KIAA1614*<br>(Chr. 1q25.3)	0	0.0001759	0.00315	0.0015	0.0036	Non-deleterious<br>0.001	Benign<br>0.000	0.84<br>Tolerated	Polymorphism<br>1			Adrenal gland
4	NM_002838:c.1498A>G<br>p.(S500G)	*PTPRC*<br>(Chr. 1q31.3)	0	0	0	0	0	Non-deleterious<br>1.689	Benign<br>0.009	Damaging<br>0.02	Polymorphism<br>1	Severe combined immunodeficiency (#608971)	AR	Brain
5	NM_175922:c.463C>T<br>p.(Q155X)	*PRR18*<br>(Chr. 6q27)	0.0006173	0.0005204	0	0.0005	0	1% most deleterious<br>23.4	−	−	Disease causing<br>1			Brain
6	NM_005299:c.83G>A<br>p.(G28D)	*GPR31*<br>(Chr. 6q27)	0	0.0009903	0.00123	0.0015	0.0036	1% most deleterious<br>24.4	−	Damaging<br>0	Disease causing<br>1			Testis, small intestine, spleen
7	NM_006024:c.1853A>G<br>p.(E618G)	*TAX1BP1*<br>(Chr. 7p15.2)	0	0.0004489	0.00263	0.0015	0	1% most deleterious<br>23.0	Benign<br>0.017	Damaging<br>0.02	Disease causing<br>1			Adrenal gland, bladder, esophageal mucosa
8	NM_182898:c.326T>A<br>p.(M109K)	*CREB5*<br>(Chr. 7p15.1)	0	0	0	0.0002	0	1% most deleterious<br>32.0	Benign<br>0.000	Tolerated<br>0.26	Disease causing<br>0.997			Whole blood
9	NM_004318:c.598G>A<br>p.(E200K)	*ASPH*<br>(Chr. 8q12.3)	0	0.00005805	0.00045	0.0017	0	1% most deleterious<br>33.0	Possibly damaging<br>0.955	Tolerated<br>0.32	Disease causing<br>0.929	Traboulsi syndrome (#601552)	AR	Adipose, adrenal gland, breast-mammary tissue
10	NM_147156:c.235G>A<br>p.(G79S)	*SGMS1*<br>(Chr. 10q11.23)	0	0.00005798	0.00395	0.0027	0.00719	1% most deleterious<br>23.7	−	Tolerated<br>0.32	Disease causing<br>1			Brain, skin, thyroid, uterus, lung
11	NM_078470:c.794A>G<br>p.(H265R)	*COX15*<br>(Chr. 10q24.2)	0	0.0001160	0.00265	0.0005	0	Non-deleterious<br>2.4	Benign<br>0.418	Tolerated<br>0.21	Disease causing<br>0.999	Cardioencephalomyopathy (#615119)<br>Leigh syndrome (#256000)	AR	Adrenal gland, bladder, ovary, pituitary
12	NM_001174084:c.1498G><br>A	*POLL*<br>(Chr. 10q24.32)	0	0.0002320	0	0.0005	0.0036	1% most deleterious<br>22.6	Probably damaging<br>1.000	Damaging<br>0.01	Disease causing<br>1			Testis, ovary, pituitary, prostate, thyroid
13	NM_007183:c.2017C>T<br>p.(R673C)	*PKP3*<br>(Chr. 11p15.5)	0.004316	0.003250	0	0.001	0	Deleterious<br>18.29	Probably damaging<br>1.000	Tolerated<br>0.27	Disease causing<br>1			Esophagus-mucosa, skin, vagina
14	NM_005961:c.2231+1G>A<br>(Splice donor mutation)	*MUC6*<br>(Chr. 11p15.5)	0	0	0	0.0002	0	1% most deleterious<br>26.8	−	−	−			Stomach
15	NM_001005172:c.302C>T<br>p.(A101V)	*OR52K2*<br>(Chr. 11p15.4)	0	0	0	0	0	Non-deleterious<br>0.007	Benign<br>0.000	Tolerated<br>0.34	Polymorphism<br>1			Spleen, lung, ovary
16	NM_001101389:c.379G>A<br>p.(A127T)	*CLDN25*<br>(Chr. 11q23.2)	0.003704	0.0073	0.00454	0.0027	0	1% most deleterious<br>36.0	Benign<br>0.409	Tolerated<br>0.15	Polymorphism<br>1			Pituitary
17	NM_001113378:c.2627A><br>G	*FANCI*<br>(Chr. 15q26.1)	0	0.00009775	0.00191	0.0017	0.0036	1% most deleterious<br>25.6	Possibly damaging<br>0.835	Tolerated<br>0.33	Disease causing<br>0.991	Fanconi anemia (#609053)		Testis
18	NM_198525:c.2571C>G<br>p.(S857R)	*KIF7*<br>(Chr. 15q26.1)	0	0.00005814	0.00322	0.002	0.00719	1% most deleterious<br>22.7	Benign<br>0.023	Tolerated<br>0.73	Polymorphism<br>0.998	Al-Gazali-Bakalinova Syndrome (#607131) Hydrolethalus Syndrome 2 (#614120) Acrocallosal Syndrome (#200660)	AR	Ovary, uterus, fallopian tube, artery, ectocervix
19	NM_145728:c.4602G>T<br>p.(M1534I)	*SYNM*<br>(Chr. 15q26.3)	0	0.0001160	0.00046	0	0	Deleterious<br>17.5	−	Damaging<br>0	Polymorphism<br>0.567			Esophageal muscularis, gastroesophageal
20	NM_002972:c.1610C>G<br>p.(T537S)	*SBF1*<br>(Chr. 22q13.33)	0	0.0004064	0.00575	0	0.00719	1% most deleterious<br>24.2	Benign<br>0.000	Tolerated<br>0.82	Polymorphism<br>1	Charcot-Marie-Tooth disease type4B3 (#615284)	AR	Testis, thyroid, brain

Shown are non-synonymous rare variants with minor allele frequencies ≤ 0.01 in the four public databases and in-house data obtained from 139 control subjects.

These variants have been selected as an autosomal-dominant or an X-linked recessive model; no variant has been identified in X-linked genes.

The URLs utilized are as follows; *in silico* analyses were performed by using the default parameters.

1) gnom AD (Genome Aggregation Database): http://gnomad.broadinstitute.org/

2) HGVD (Human Genetic Variation Database): http://www.hgvd.genome.med.kyoto-u.ac.jp/

3) 2KJPN (Whole-genome sequences of 2,049 healthy Japanese individuals and construction of the highly accurate Japanese population reference panel): https://ijgvd.megabank.tohoku.ac.jp/

4) CADD (Combined Annotation–Dependent Depletion): http://cadd.gs.washington.edu/score (Current version: 1.3, GRCh37/hg19); PHRED scores of > 10–20 are regarded as deleterious, and those of > 20 indicates the 1% most deleterious.

5) Polyphen-2 Hum Var: http://genetics.bwh.harvard.edu/pph2/ (Current version: 2.2.2, GRCh37/hg19); HumVar scores were evaluated as 0.000 (most probably benign) to 1.000 (most probably damaging).

6) SIFT (Sorting Intolerant From Tolerant): http://sift.jcvi.org/ (Current version: Aug. 2011; GRCh37/Ensembl 63)); Scores of ≤0.05 and those > 0.05 are assessed as damaging and tolerated, respectively.

7) MutationTaster: http://www.mutationtaster.org/ (MutationTaster2, GRCh37/Ensembl 69); Alterations are classified as disease causing or polymorphisms, and the scores indicate the probabilities of predictions.

8) OMIM (Online Mendelian inheritance in man): http://omim.org/

9) UCSC genome browser: https://genome.ucsc.edu/

No variant has been found in the known or candidate autosomal dominan or X-linked recessive genes for DSD[1,2].

References

1. Achermann, J.C. & Hughes, I.A. Pediatric disorders of sex development. In: Melmed S, Polonsky KS, Larsen PR, Kronenberg HM, eds. Williams textbook of endocrinology. 13th ed. Philadelphia: Elsevier 893–963 (2016).

2. Eggers, S. *et al*. Disorders of sex development: insights from targeted gene sequencing of a large international patient cohort. *Genome Biol.* 17, 243 (2016).

Supplementary Table S2. Primers utilized in this study

	Forward (5' → 3')	Reverse (5' → 3')
<*AR* sequencing>		
Exon 1a	GCCTGTTGAACTCTTCTGAGC	CTGTGAAGGTTGCTGTTCCTC
Exon 1b	CACAGGCTACCTGGTCCTGG	CTGCCTTACACAACTCCTTGGC
Exon 1c	CCACTTCCTCCAAGGACAATTAC	CGGGTTCTCCAGCTTGATGCG
Exon 1d	CAGAGTCGCGACTACTACAACTT	CTGGGATAGGGCACTCTGCTCA
Exon 1e	GACTTCACCGCACCTGATGTGTG	CAGAACACAGAGTGACTCTGCCC
Exon 2	CTGCAGGTTAATGCTGAAGACC	TAAGTTATTTGATAGGGCCTTGCC
Exon 3	GTTTGGTGCCATACTCTGTCCAC	CTGATGGCCACGTTGCCTATGAA
Exon 4	GAGTTTAGAGTCTGTGACCAGG	GATCCCCCTTATCTCATGCTCC
Exon 5	CAACCCGTCAGTACCCAGACTGA	GCTTCACTGTCACCCCATCACCA
Exon 6	CTCTGGGCTTATTGTAAACTTCC	TCCAGGAGCTGGCTTTTCCCTA
Exon 7	CTTTCAGATCGGATCCAGCTATCC	CTCTATCAGGCTGTTCTCCCTGAT
Exon 8	GAGGCCACCTCCTTGTCAACC	GGAACATGTTCATGACAGACTG
<*NR5A1* sequencing>		
Exons 2+3	GCACAGAGAGGGGATTACGCG	ACTATCCCCTCAGCCCCTCTC
Exon 4-1	TCTGTGGTGGGGCAGCATGG	ATGGCACGGCCAGGAAAGGC
Exon 4-2	TGGGCCACTGGGCGACTTTG	TCGGGCTAAGGCTTGGGCAG
Exon 5	GTGAGAGGAAGGTCCCTGGAC	GGGCCCTGAATCCTGGAAGTG
Exon 6	GCACCTCCAATCCATGCCCTC	TCCTTCGTGGCCACTCTGGC
Exon 7	TGTCTTTGATGGTCATAGGGAAGGTGG	ACCTTCCCAAACACACAGTGTCAGAAC
<*MAMLD1* sequencing>		
Exon 2	CTGTGATTCACGGGGTCAGT	CGTTCAAGTTTAAGATTAGAAGCA
Exon 3	CACTGAGCTGGTGCAGGTAA	GCCTCAGTTTCCCCACTGTA
Exon 4-1	CTCTCTTCTCCTCTTCTCTTCTCT	GGTGAGCTCCTCTAGCAGCTCTTG
Exon 4-2	GCCGGCTGTAGACCAGGAGC	GGGCGTGATGCCACTGAGGC
Exon 4-3	GTCGCTCTGCCCCCCTTACC	GAATGGAGCTCTGAGGGCCG
Exon 4-4	GCTCTCACTCAACAGCCGCAG	GGCTCCGTGGCTCCTGGGCTG
Exon 4-5	CATGATCATGCAGCAGGGGATGGCAAGC	CCAAAACAAAAGAACACAGCCAGATATG
Exon 4-6	CAACGCAGGCCTCCTCAG	AGTTAGGTGACTTGAACAGGCA
Exon 5	TAGGACACGGCAGGCCACCTG	AGCCAGCTGATCCAGCTCTGG
Exon 6	TTTGTGGCCAAGCAGCTGATG	GACTGTGCCCCGTTGACAGAT
Exon 7	CTAGGTCCCCACGCAGCGATC	ACCCCTTGCCCACCCCTTTGG
<*SRD5A2* sequencing>		
Exon1	GAGGTGGGAGGCAGGATG	GATGAGGTCCTGGGGGAG
Exon2	CATCCTCCTGGAGAAGATAATTTG	GTTTAAACAATCTCTCTGGCTACC
Exon3	CCACTTTCTGCCACGTCTTA	GTTTGCAGGGGAAGTCAAGA
Exon4	CCTTCTCCCCAAGAGGATTC	TACAAGCCCAGCAAGTCAGA
Exon5	ATATTGCACAGCCATCACCA	AGCAGACACCACTCAGAATCC
<*AR* analysis>		
(CAG)n	TCCAGAATCTGTTCCAGAGCGTGC	GCTGTGAAGGTGGCTGTTCCTCAT
c.2450–42G>A	CTTTCAGATCGGATCCAGCTATCC	CTCTATCAGGCTGTTCTCCCTGAT
<*AR* expression analysis>		
P1/P2	CGATCCTTCACCAATGTCAA	CAGGTCAAAAGTGAACTGATGC
PW/PC	TTCAGCATTAGCATGCTTCC	CTCTCGCAATAGGCTGCACGG
PM/PC	TTCAGCATTATTCCAGTGGA	CTCTCGCAATAGGCTGCACGG
